# Combined analyses of mRNA and miRNA transcriptome reveal the molecular mechanisms of theca cells physiological differences in geese follicular selection stage

**DOI:** 10.1016/j.psj.2024.104402

**Published:** 2024-10-10

**Authors:** Xinyue Hu, Hengli Xie, Xi Zhang, Yueyue Lin, Shenqiang Hu, Jiwei Hu, Hua He, Liang Li, Hehe Liu, Jiwen Wang

**Affiliations:** State Key Laboratory of Swine and Poultry Breeding Industry, College of Animal Science and Technology, Sichuan Agricultural University, Chengdu, Sichuan, 611130, PR China

**Keywords:** miRNA-seq, mRNA-seq, theca cell, goose, follicle selection

## Abstract

In avian, follicular selection is a key molecular event that can determine avian egg production. Theca cells (**TC**) are the main components of follicles, the molecular mechanisms about TCs physiological differences during follicle selection stage are still unclear. This study revealed significant differences in proliferation, apoptosis, lipid synthesis, and steroid secretion levels between prehierarchical theca cells (**phTC**) and hierarchical theca cells (**hTC**) of Tianfu meat-type geese. A total of 1,559 differentially expressed genes (**DEG**) and 71 differentially expressed miRNAs (**DEM**) were identified between phTCs and hTCs, respectively. Functional enrichment analysis results showed that 143 DEGs were enriched in the pathways related to cell proliferation/apoptosis and lipid/steroid metabolism. Protein-protein interaction (**PPI**) network results indicated the 143 DEGs have functional interactions. Additionally, the predicted target genes of 71 DEMs were jointly analyzed with the above 143 DEGs, and the results showed that 15 DEMs and 17 DEGs with targeted relationships were found. Among them, *miR-202-5p* was significantly down-regulated both in hTCs and hierarchical theca layers, and target prediction results showed that *miR-202-5p* may affect TCs proliferation/apoptosis by targeting *CHPT1* to regulate the expression levels of *CCN1*/*FOXO3*; meanwhile, may affect TCs lipid/steroid metabolism and proliferation/apoptosis by targeting *CHPT1* to regulate the expression levels of *p53*/*ABCA1*/*SREBP*-2. This study provides new insights into the regulatory mechanisms of TCs physiological differences during goose follicle selection.

## INTRODUCTION

Avian follicles follow a special hierarchy development system, according to the diameter and appearance, follicles can be divided into prehierarchical and hierarchical follicles (Lovell, et al., 2003). In chicken, prehierarchical follicles included small white follicles (SWFs, 2–4 mm in diameter), large white follicles (LWFs, 4–6 mm in diameter), and small yellow follicles (SYFs, 6–8 mm in diameter) (Johnson, 2015). The process that a SWF selected to initiate rapid growth and differentiation is follicle selection (Johnson and Woods, 2009), the selected SWF will enter ovulation after depositing a large amount of yolk under FSH stimulation (Johnson, 2012), while unselected SWFs are required to maintain a state of undifferentiated growth to provide the possibility of being selected for the next selection cycle (Johnson, 2015). However, more than 90% of follicles are not selected and eventually become atresia (Matsuda, et al., 2012). Follicle selection is a decisive step in determining the number of hierarchical follicles, even the eventual egg production (Johnson, 2012). Unlike chickens and ducks, which produce 200 to 300 eggs annually (Johnson, et al., 2015), most breeds of geese produce only 20 to 40 eggs annually. It is speculated that this difference may be caused by follicle selection.

Studies to date have shown that theca cells (**TC**) are important components of follicles, which can maintain follicular physical structure, follicular diameter increases, and steroid hormone continuous secretion (Magoffin, 2005). In TCs, cholesterol (**CH**) is transported and cleaved by STAR and CYP11 enzymes (Farkash, et al., 1986; Stocco, 2001), then through the action of 3βHSD (Cherradi, et al., 1994), 17βHSD, CYP17 (Cherradi, et al., 1994), and CYP19 (Wang and Gong, 2017) enzymes, progesterone, androgen, and estrogen are synthesized, respectively. Moreover, in the prehierarchical follicles whose granulosa cells have not yet differentiated in avian, the syntheses of steroid hormones in follicles are almost entirely responsible by TCs (Johnson and Woods, 2009). Otherwise, when prehierarchical follicles were selected to enter the hierarchical follicle stage, the number of TCs would increases (Kim, et al., 2016), and the TCs will metabolize abundant fatty acids for energy production (Dong, et al., 2014; Foxcroft and Hunter, 1985; Hunter, et al., 2004). Therefore, the physiological and functional changes of TCs might are important reflections of follicle selection, and understanding the molecular mechanisms regulating the physiological functions of TCs during goose follicle selection stage could provide important theoretical references for improving goose egg production.

MicroRNAs (**miRNAs**) are a class of non-coding single strandedness molecules with a length of 21 to 25 nt, miRNAs participate in post transcriptional regulation of genes in animals and plants (Glazov, et al., 2008). It has been found that miRNAs can participate in the regulation of multiple physiological processes through targeted genes, and play an important role in the regulation of ovarian function (Maalouf, et al., 2016). Growing studies demonstrate that miRNAs are express in the ovaries of mammals and avian (Kang, et al., 2013; Maalouf, et al., 2016; Wang, et al., 2018; Zhang, et al., 2019), they are involved in almost all ovarian biological processes, including follicular development, atresia, ovulation, and degeneration. In avian, differentially expressed miRNAs (**DEM**) have been demonstrated in sexually mature and immature chicken ovaries (Kang, et al., 2013), ovaries of hens with high and low egg production (Wu, et al., 2017), ovaries of laying and broody geese (Xu, et al., 2014), healthy and atretic follicles (Liu, et al., 2018), as well as the follicular theca and granulosa layer of goose follicles in 3 developmental stages (Li, et al., 2019). However, there is limited data on the expression and role of miRNAs in follicular TCs during follicular development. Therefore, the present study aimed to detect the cell viability and proliferation/apoptosis rate of prehierarchical theca cells (**phTCs**) and hierarchical theca cells (**hTCs**) of Tianfu meat-type geese, as well as the lipid deposition and steroid hormone secretion in phTCs and hTCs. Then, mRNA and miRNA transcriptome were used to study the differences of miRNA/mRNA expression profiles to reveal the molecular mechanisms of TCs physiological differences in goose follicle selection stage.

## MATERIALS AND METHODS

### Animals

The healthy maternal line of Tianfu meat-type geese, which were at the peak of egg-laying period, were used for this study. These geese were raised in the Sichuan Agricultural University Waterfowls Breeding Farm (Ya'an, Sichuan, China) under natural light and temperature conditions, as well as free food and water. According to the egg laying records, 3 geese that at 35 to 45 wk of age and the frequency of laying eggs were 1 egg per 2 days were selected. After the worker touched the abdomen to determine the presence of a hard-shelled egg in the oviduct (the presence of a hard-shelled egg in the oviduct indicates that an egg will be laid in the near future, at this time, there is a high probability of F1 follicle formation, which is convenient for the differentiation of F1, F2, F3, F4, F5, and F6 follicles), the 3 geese were used to collect follicles for each experiment. As shown in Supplementary Figure S1, according to the diameter, follicles are classified into prehierarchical (8–10 mm) and hierarchical follicles (F6–F1, F1 > F2 > F3 > F4 > F5 > F6 in diameter).

### Theca Cell Culture

The theca layers of prehierarchical (8–10 mm) and hierarchical (F4–F2) follicles were separated according to previous methods (Gan, et al., 2017). The theca layers at different stages were placed into 5 mL centrifuge tubes respectively, and were cleaned with Phosphate Buffered Saline (PBS, Gibco, Shanghai, China). Then, type I collagenase (Gibco, Shanghai, China) with a concentration of 0.5% was used to digest theca layers. The digestion was terminated with cold PBS, and the scattered TCs were filtered through a 200-mesh sieve and separated by centrifugation at 1,000 RPM for 10 min at room temperature. Subsequently, the TCs were resuspended in Dulbecco's Modified Eagle's Medium/Nutrient Mixture F12 (DMEM/F12, Gibco, Shanghai, China) containing 10% Fetal Bovine Serum (**FBS**, Gibco, Shanghai, China). Finally, the TCs in suspensions were seeded in 12-well or 96-well plates with a density of 5 × 10^5^ cells/mL to culture at constant temperature of 37°C, 5% CO_2_, 95% air, and saturated humidity. After 6 h of culture, the medium was changed to remove the non-adherent TCs.

### Detection of TCs Viability

In this assay, CCK8 assay kit (Vazyme, Nanjing, China) was used to detect the viability of phTCs and hTCs. 10 µL of CCK8 solutions were added to the 96-well plates cultured TCs, after incubated at 37°C for 4 h. The absorbance value at 450 nm was detected by microplate reader to reflect cell viability. The experiment was repeated 3 times.

### Detection of TCs Proliferation

Cell-Light EdU Apollo567 in Vitro Kit (RiboBio, Guangzhou, China) was used to detect the proliferation rate of phTCs and hTCs. The cell proliferation rate was calculated as the ratio of the number of EdU-incorporated cells to the number of Hoechst33342 staining cells. At least 500 cells were counted every group. The experiment was repeated 3 times.

### Detection of TCs Apoptosis

To detect the cell apoptosis rate, phTCs and hTCs were stained by the Annexin V-FITC/PI Apoptosis Detection Kit (Vazyme, Nanjing, China) according to the manufacturer's instructions. BD Accuri C6 Flow cytometers (BD Biosciences, NJ) were used to quantify apoptotic cells. The FlowJo software (version 10.8.1) was used to analyze early apoptosis rate, late apoptosis rate, and live cell rate, and the apoptosis rate was the sum of early and late apoptosis rates (Chen, et al., 2020). The experiment was repeated 3 times.

### Detection of Triglyceride and CH Contents in TCs

Radioimmunoprecipitation assay (**RIPA**) buffer (Thermo Fisher Scientific, Waltham, MA) and phenylmethanesulfonylfluoride fluoride (**PMSF**) were used to collect phTCs and hTCs. Then, using the triglyceride (**TG**) and total CH ELISA Assay Kits (Nanjing Jiancheng Bioengineering Institute, Nanjing, China) to determine the concentrations of TG and CH in phTCs and hTCs according to the manufacturer's instructions. TG and TC contents were evaluated by the ELISA reader at 450 nm. The experiment was repeated 3 times.

### Detection of Steroid Hormone Secretions of TCs

The cell culture medium supernatant was collected to determine the secretions of progesterone, estradiol, and testosterone in phTCs and hTCs by using Goose progesterone, estradiol, and testosterone ELISA Kit (Nanjing Jiancheng Bioengineering Institute, Nanjing, China) according to the manufacturer's instructions. progesterone, estradiol, and testosterone contents were evaluated by the ELISA reader at 450 nm. The experiment was repeated 3 times.

### RNA Extraction, Library Preparation, and Sequencing

The total RNA of phTCs (8–10 mm, n = 3) and hTCs (F4–F2, n = 3) were extracted by TRIzol Reagent according to the manufacturer's instructions (Invitrogen, Waltham, CA). RNA integrity was analyzed on the Agilent 2100 Bioanalyzer System (Agilent Technologies, Palo Alto, CA). The 6 RNA samples were used for library construction, and mRNA and small RNA libraries were sequenced by illumina platform.

### mRNA-seq and Bioinformatics Analysis

Clean reads were obtained after filtering low-quality reads using standard quality control from the FastaQC software. Clean reads were mapped to the reference genome of Sichuan White goose (data being published) by HISAT2 (version 2.2.1) software (Kim, et al., 2019). Subsequently, the expression level of each transcript was calculated through featureCounts (version 1.6.0) (Liao, et al., 2014). DESeq2 package (version 1.34.0) was used to identify differentially expressed genes (DEGs) with the conditions of |log_2_ (Fold change)| ≥ 1 and *P* value < 0.05. KOBAS 3.0 online software was used to perform functional enrichment analysis of DEGs (Bu, et al., 2021).

### miRNA-seq and Bioinformatics Analysis

Cutadapt (version 3.7) software was used for quality control to remove adapters and low-quality reads, and the sequences who larger than 30 nt and smaller than 18 nt were filtered out. The remaining reads were aligned with miRBase (version 21) database to identify known miRNAs, and novel miRNAs were predicted by mirdeep2 software (version 0.1.3). DESeq2 package (version 1.34.0) was used to identify DEMs with the conditions of |log_2_ (Fold change)| ≥ 1 and *P* value < 0.05. The target genes of DEMs were predicted by RNAhybrid (version 2.2), TargetScan (version 7.2), and PITA software.

### Quantitative Real-Time PCR Validation of mRNA/miRNA

RT-qPCR were used to confirm the expression levels of mRNAs and miRNAs in this study, and 4 DEGs and 1 DEMs were selected for validation. As the manufacturer's instructions, total RNAs were isolated from 3 phTCs and hTCs samples. Subsequently, the miRNA 1st Strand cDNA Synthesis Kit (by tailing A) (Vazyme, Nanjing, China) was used to convert miRNA into cDNA, while the HiScript II 1st Strand cDNA Synthesis Kit (Vazyme, Nanjing, China) was used to convert mRNA into cDNA. All primers, including the reference genes named *U6* and *GAPDH*, used in this study were shown in Supplementary Table S1. Then, qPCR was performed by using the 2 × SYBR Premix Ex Taq II (TaKaRa, Dalian, China) in each sample. Finally, the Ct value of each mRNA/miRNA were normalized with *GAPDH* or *U6* using the 2^–ΔΔCt^ method (Schmittgen and Livak, 2008).

### Statistical Analysis

Data was analyzed by using SPSS 27.0 software (IBM, Chicago, IL). Pictures were plotted by using GraphPadPrism (version 5.0) software. Results were expressed as mean ± standard deviation. Independent sample t-test was used for comparison between phTCs and hTCs groups, and *P* < 0.05 was considered statistically significant.

## RESUILS

### Differences in Physiological Function Between phTCs and hTCs

**Figure 1:**
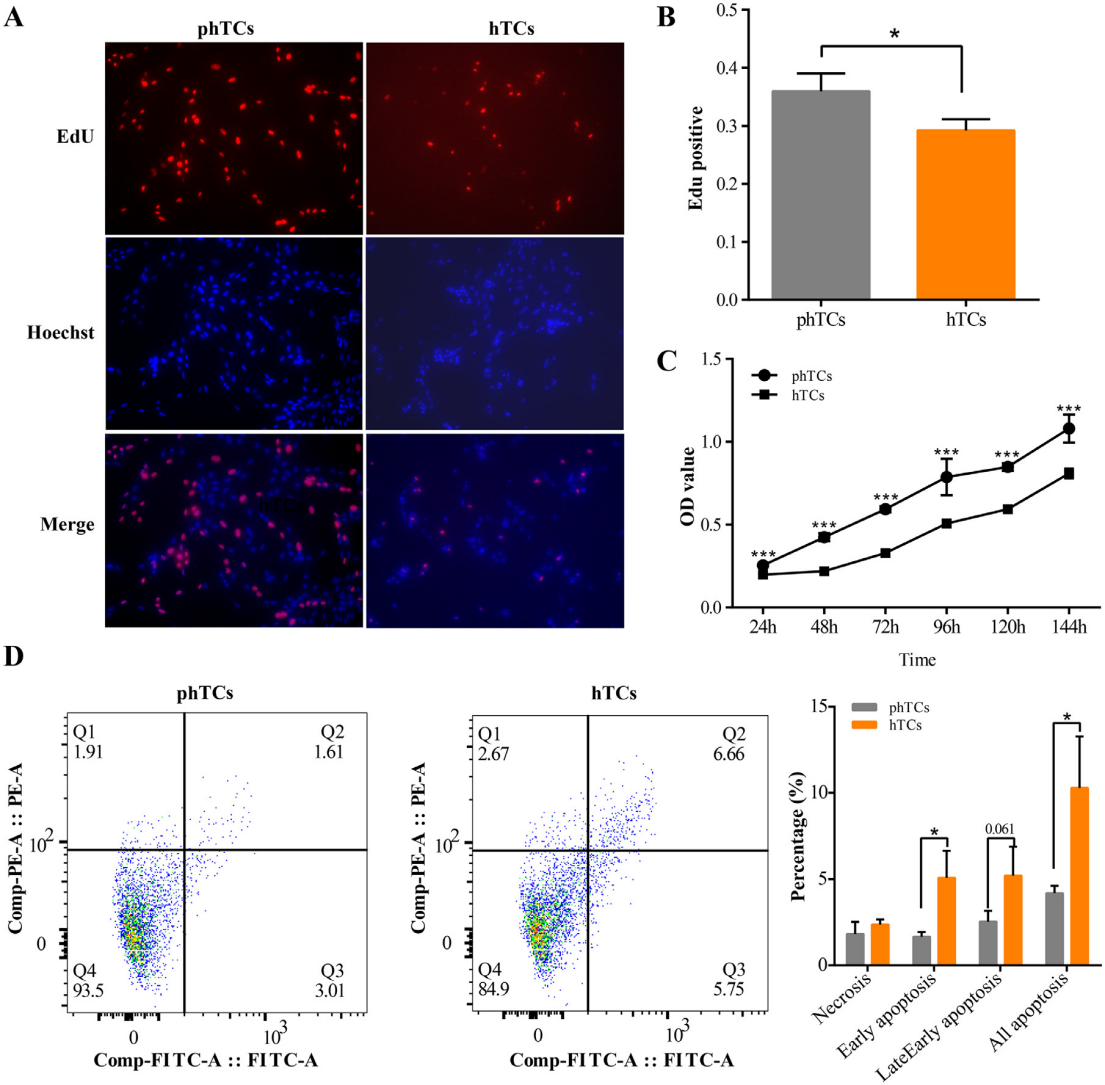
phTCs and hTCs proliferation, viability and apoptosis levels. (A) EdU staining was used to detect proliferation rate of phTCs and hTCs. (B) EdU incorporation rate was calculated. (C) CCK8 was used to detect phTCs and hTCs viability. (D) Apoptosis rate of phTCs and hTCs were detected by flow cytometer. * indicates *P* < 0.05, ** indicates *P* < 0.01, *** indicates *P* < 0.001. Abbreviations: Edu: 5-Ethynyl -2′- deoxyuridine; phTCs: prehierarchical theca cells; hTCs: hierarchical theca cells; CCK8: cell counting kit-8.

**Figure 2:**
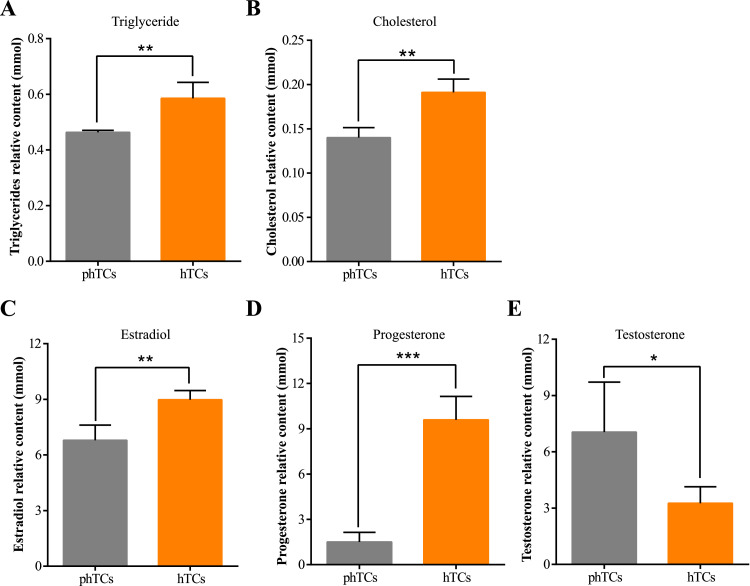
Determination of intracellular lipids and hormone contents in phTCs and hTCs. Intracellular TG (A) and CH (B) contents in phTCs and hTCs. Secretion level of Estrogen (C), Progesterone (D), and Testosterone (E) in phTCs and hTCs. * indicates *P* < 0.05, ** indicates *P* < 0.01, *** indicates *P* < 0.001. Abbreviations: TG: triglycerides; CH: cholesterol; phTCs: prehierarchical theca cells; hTCs: hierarchical theca cells.

As showed in Figure 1, the proliferation rate of phTCs was significantly higher than that in hTCs (Figures 1A and 1B, *P* < 0.001), while the apoptosis rate of phTCs was significantly lower than that of hTCs (Figure 1D, *P* < 0.05). In addition, the cell viability of phGCs was significantly higher than hTCs during the culture period (Figure 1C, *P* < 0.05). As showed in Figure 2, the TG (Figure 2A) and CH (Figure 2B) contents in hTCs were significantly higher than that in phTCs (*P* < 0.01), and the Estrogen (Figure 2C) and Progesterone (Figure 2D) contents were significantly higher than that in phTCs (*P* < 0.01) too, while the Testosterone (Figure 2E) contents showed the opposite trend between 2 groups (*P* < 0.05).

### Overview of Sequencing Data

As shown in Supplementary Table S2, a total of 136,405,851 raw reads were obtained by mRNA sequencing, and the average ratios of Q20 and Q30 were 95.78% and 88.22%, after removing the adaptors and low-quality reads, 132,619,605 clean reads were obtained, and the average mapping rate of clean reads was 93.51%. In addition, as shown in Supplementary Table S3, a total of 72,453,425 raw reads by miRNA sequencing were obtained. The average values of Q20 and Q30 were 96.79% and 87.67% respectively. After the low-quality reads were removed, a total of 64,867,546 clean reads were obtained.

### Functional Analysis of DEGs Between phTCs and hTCs

**Figure 3:**
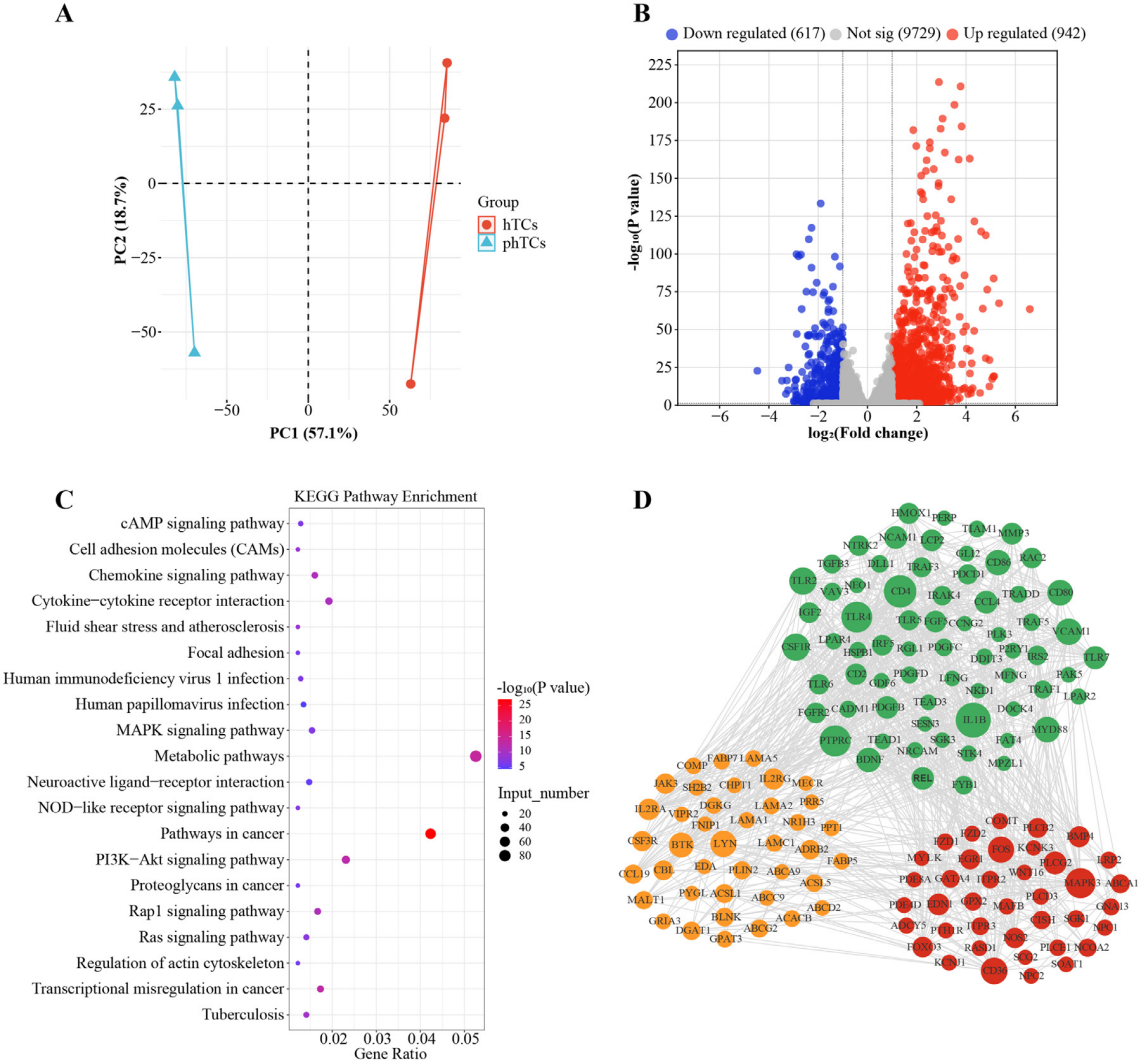
RNA-seq of phTCs and hTCs. (A) mRNA PCA analysis in phTCs and hTCs groups. (B) Volcano plot of mRNA. (C) KEGG pathways enriched by DEGs. (D) PPI networks of DEGs. Genes related to cell proliferation/apoptosis are shown in green, lipid metabolism related genes are shown in orange, and steroid metabolism related genes are shown in red. Abbreviations: PCA: principal component analysis; phTCs: prehierarchical theca cells; hTCs: hierarchical theca cells; KEGG: Kyoto Encyclopedia of Genes and Genomes; DEGs: differentially expressed genes; PPI: protein-protein interaction.

PCA result showed that the samples within each group were relatively concentrated, while the samples between groups were relatively scattered (Figure 3A). A total of 1,559 DEGs were obtained, of which 617 were down-regulated and 942 were up-regulated (Figure 3B). Otherwise, KEGG functional enrichment analysis showed that 170 KEGG pathways were significantly enriched by DEGs (*P* < 0.05), of which Rap1 signaling pathway, MAPK signaling pathway, Ras signaling pathway, Cell adhesion molecules (CAMs) pathway, and Focal adhesion pathways are related to cell proliferation/apoptosis; cAMP signaling pathway, PI3K-Akt signaling pathway, fatty acid biosynthesis, and fatty acid metabolism pathways are related to cellular lipid metabolism; and Cushing syndrome, Parathyroid hormone synthesis, secretion and action, Estrogen signaling pathway, Cholesterol metabolism, and Aldosterone synthesis and secretion pathways are related to cellular steroid metabolism (Figure 3C). Further, protein-protein interaction (**PPI**) network results indicated that DEGs who are involved in proliferation/apoptosis (65 DEGs), steroid synthesis (39 DEGs), and lipid metabolism (40 DEGs) related pathways had functional interactions (Figure 3D).

### Functional Analysis of DEMs Between phTCs and hTCs

**Figure 4:**
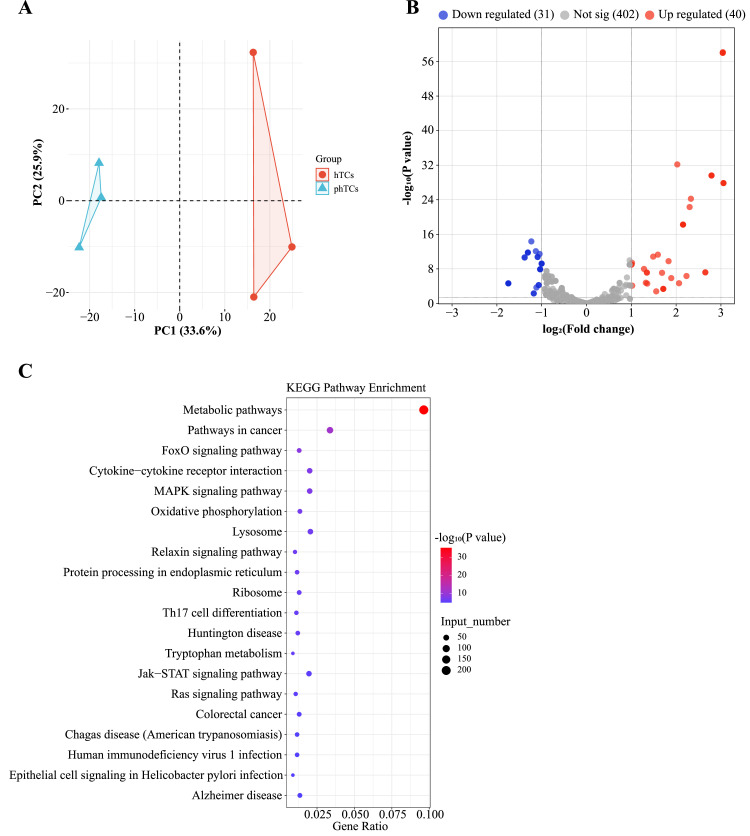
miRNA-seq of phTCs and hTCs. (A) miRNA PCA analysis in phTCs and hTCs groups. (B) Volcano plot of DEMs. (C) KEGG pathways enriched by DEMs target genes. Abbreviations: phTCs: prehierarchical theca cells; hTCs: hierarchical theca cells; KEGG: Kyoto Encyclopedia of Genes and Genomes; DEMs: differentially expressed miRNAs.

PCA result showed the samples within each group were relatively concentrated, while the samples between groups were relatively scattered (Figure 4A). A total of 465 known miRNAs and 8 new miRNAs were discovered, and 71 DEMs were identified between phTCs and hTCs, of which 31 were down-regulated and 40 were up-regulated (Figure 4B, Supplementary Table S4). In addition, 2,139 target genes of the 71 DEMs were predicted by using PITA, TargetScan, and RNAhybrid software. Functional enrichment analysis showed that the 2,139 genes were significantly enriched in 52 pathways (*P* < 0.05), including MAPK signaling pathway, FOXO signaling pathway, and Ras signaling pathways associated with cell proliferation/apoptosis; Fatty acid degradation, Fatty acid biosynthesis, PI3K-Akt signaling pathway, and Triglyceride metabolism pathways associated with lipid metabolism; and Parathyroid hormone synthesis, secretion and action, Cushing syndrome, Cholesterol metabolism, Thyroid hormone signaling pathway, Estrogen signaling pathway, and Steroid hormone biosynthesis pathways associated with steroid metabolism (Figure 4C).

### Combined Analysis of miRNA and mRNA Sequencing

**Figure 5:**
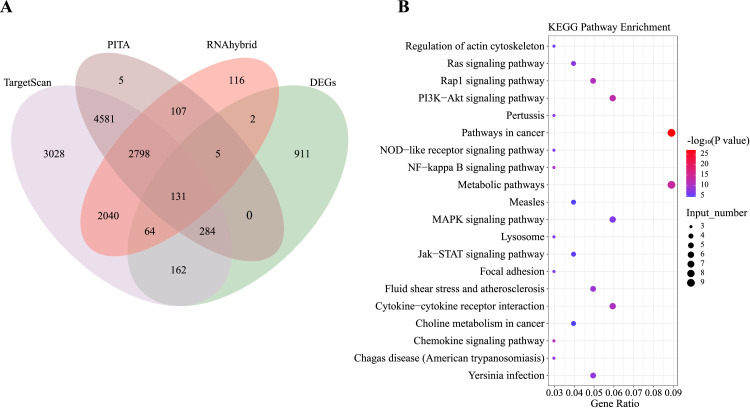
Combined analysis of miRNA and mRNA sequencing. (A) Venn diagram of predicted target genes of DEMs. (B) KEGG pathways enriched by DEGs who were the target genes of DEMs. Abbreviations: DEMs: differentially expressed miRNAs; DEGs: differentially expressed genes; KEGG: Kyoto Encyclopedia of Genes and Genomes.

To further investigate the regulatory role of miRNAs, target genes predicted by DEMs were jointly analyzed with DEGs, and a total of 131 common genes were identified (Figure 5A). KEGG functional enrichment analysis showed that the 131 genes were significantly enriched in 105 pathways (*P* < 0.05), of which MAPK signaling pathway, FOXO signaling pathway, and Ras signaling pathways are related to cell proliferation and apoptosis; PI3K-Akt signaling pathway, fatty acid metabolism, fatty acid synthesis, and glycerophospholipid metabolism pathways are related to lipid metabolism; and metabolism pathway is related to steroid metabolism (Figure 5B).

### Screening of key DEMs and Target Genes Between phTCs and hTCs

**Figure 6:**
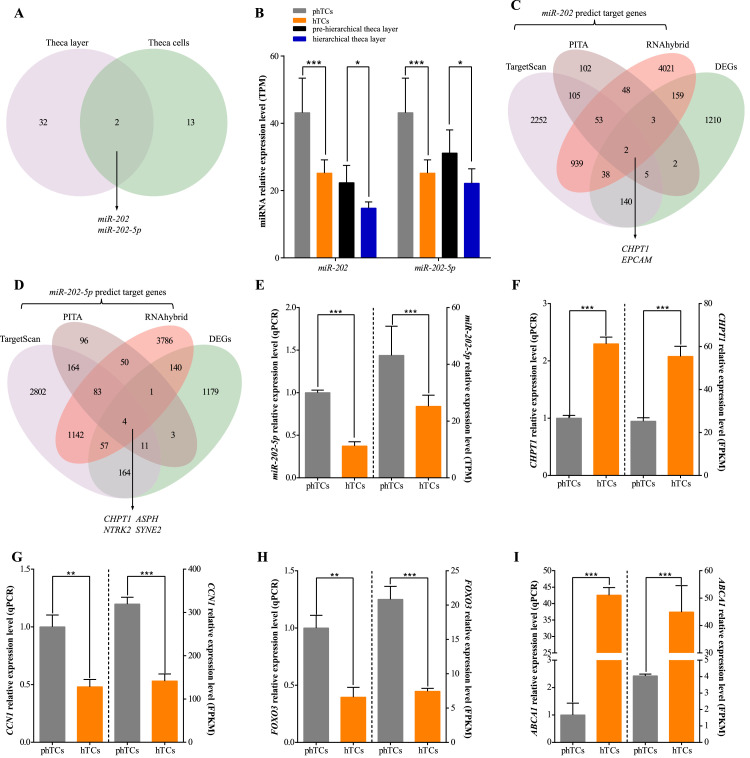
Screening of key miRNAs and DEGs. (A) Veen plot of key DEMs in goose theca layers and TCs. (B) The TPM of *miR-202* and *miR-202-5p* in prehierarchical/hierarchical theca layers and phTCs/hTCs. (C) Venn diagram of *miR-202* target genes. (D) Venn diagram of *miR-202-5p* target genes. qPCR and transcriptome sequencing results of *miR-202-5p* (E), *CHPT1* (F), *CCN1* (G), *FOXO3* (H), and *ABCA1* (I) in phTCs and hTCs. ** indicates *P* < 0.01, *** indicates *P* < 0.001. Abbreviations: DEMs: differentially expressed miRNAs; DEGs: differentially expressed genes; TCs: theca cells; TPM: Transcripts Per Million; phTCs: prehierarchical theca cells; hTCs: hierarchical theca cells; qPCR: quantitative polymerase chain reaction; *ABCA1*: ATP binding cassette subfamily A member 1; *CHPT1*: Choline phosphotransferase 1; *CCN1*: Cellular communication network factor 1; *FOXO3*: Forkhead box O3.

Based on the results of functional analysis, a total of 15 DEMs and its 17 target DEGs related to cell proliferation/apoptosis and lipid/steroid metabolism were screened (Supplementary Table S5). In order to further screen out the key miRNAs that stably regulate the physiological function of TCs both in vivo and vitro levels, relative miRNA-seq data from theca layers of prehierarchical and hierarchical follicles in geese was downloaded and jointly analyzed with the above 15 DEMs (Li, et al., 2019). It was found that *miR-202* and *miR-202-5p* were also differentially expressed in the prehierarchical and hierarchical theca layers (Figure 6A), and *miR-202* and *miR-202-5p* were significantly up-regulated in the phTCs/prehierarchical theca layers (Figure 6B). As shown in Figures 6C and 6D, the DEGs named *EPCAM* and *CHPT1* are the target genes of *miR-202*, while DEGs named *ASPH, CHPT1, NTRK2*, and *SYNE2* are the target genes of *miR-202-5p*. In addition, the expression levels of *miR-202-5p, CHPT1, ABCA1, CCN1*, and *FOXO3* detected by qPCR were similar to the RNA-seq results (Figures 6E–6I), indicating the reliability of the sequencing data.

## DISCUSSION

TCs are an important part of the follicles, follicles will fail to ovulate if without TCs (Magoffin, 2005). Follicular development is a complex reproduction-related physiological process including cell proliferation, differentiation, and apoptosis (Adashi, 1998; Kim, et al., 2016). In this study, CCK8 and EdU results showed that the viability and proliferation capacity of phTCs were higher than hTCs significantly. Otherwise, one previous study found vast DEGs and differentially expressed circRNA between phTCs and hTCs in chicken were associated with proliferation (Shen, et al., 2020). These results indicated that the proliferation capacity of TCs might increase from the prehierarchical to hierarchical follicles. The present study found that Estradiol and Progesterone secretion capacity of hTCs was significantly higher than that of phTCs. Similar results have found in chickens, the increase of progesterone secretion during the ovulatory cycle promotes follicle maturation and ovulation (Zalanyi, 2001). In addition, steroidogenesis requires a continuous supply of CH (Toth, 1992). This point is consistent with the finding that the Estrogen, Progesterone, and CH contents in hTCs were significantly higher than that in phTCs in the present study. To further understand the molecular mechanisms regulating the physiological changes of TCs during follicle selection, transcriptome sequencing was performed on phTCs and hTCs. A total of 1,559 DEGs were obtained between phTCs and hTCs, and the results of functional enrichment analysis showed that DEGs were enriched in multiple pathways related to cell proliferation/apoptosis and lipid/steroid metabolism. Subsequently, the PPI network analysis result of DEGs enriched in pathways related to proliferation/apoptosis and lipid/steroid metabolism indicated that above 4 biological processes in TCs may interact with each other.

Several studies have shown that miRNAs play important regulatory roles in the ovarian (Lei, et al., 2010; Yao, et al., 2010; Oclon and Hrabia, 2021). However, there are few studies on the molecular mechanism of miRNA regulating the physiological function of TCs during goose follicle selection stage. In the present study, a total of 71 DEMs between phTCs and hTCs were identified. Then, the target genes of 71 DEMs were predicted, and after combined analysis the target genes with DEGs identified between phTCs and hTCs, 15 DEMs (*miR-223-3p, miR-1388a-5p, miR-455-3p, miR-455, miR-455-5p, miR-460a-5p, miR-214-3p, miR-214, miR-125-5p, miR-1416-5p, miR-221-5p, miR-202, miR-202-5p, miR-378, miR-378-3p*) were thought to regulate the proliferation/apoptosis and lipid/steroid metabolism of TCs by mediating 17 DEGs. Among the 15 DEMs, some have been validated to be associated with follicular development. For instance, *miR-378* (Xu, et al., 2011), *miR-214-3*p (Shi, et al., 2020) and *miR*-*455-3p* can affect porcine follicular development (Shen, et al., 2024). Moreover, *miR-378* and *miR-378-3p* are shown to be associated with follicular development in mouse (Sun et al., 2018; Sun et al., 2020). Notably, *miR-202-5p* not only has been shown to affect follicle maturation in goats (Ding, et al., 2020; Feng, et al., 2022), but also has been shown to regulate granulosa cell proliferation, apoptosis, and lipid/steroid metabolism in geese (Ran, et al., 2023a,b). However, further studies are needed to confirm the functionality of these DEMs.

To further screened the key miRNAs that stable regulated the physiological function of TCs both in vivo and vitro. The miRNA-seq data of prehierarchical and hierarchical theca layers were downloaded and were jointly analyzed with the 15 DEMs screened in the present study (Li, et al., 2019). Interestingly, *miR-202-5p* was found significantly down-regulated in hierarchical theca layers and hTCs. Previous studies have shown that *miR-202-5p* can affect the follicular development of black goat and geese by regulating the physiological function of granulosa cells (Feng, et al., 2022; Ran, et al., 2023a,b). However, the regulatory mechanisms of *miR-202-5p* on the physiological function of TCs has not been reported. It is well known that miRNAs participate in animal physiological processes mainly by regulating the expression of target genes at the post-transcriptional level (Bartel, 2009). In this study, it was found that *CHPT1* was significantly differentially expressed between phTCs and hTCs, and the change trend was opposite to *miR-202-5p*. Previous studies have shown that *CHPT1* is able to catalyze the acyl transfer of Long-chain fatty acid Coenzyme A conjugates to carnitine, which is an important step in the uptake and an importan of long-chain fatty acids by mitochondria (L, et al., 1998; Prip-Buus, et al., 2001; Gobin, et al., 2003). In addition, ATP released from the above process can affect AMPK enzyme activation, and influence the phosphorylation of AMPK and p53 pathways to affect cell proliferation (Zhuang, et al., 2019). Previous studies have shown that *CCN1* can affect cell migration and differentiation, which is related to the phosphorylation of AMPK too (Park, et al., 2015). *FOXO3* is an important gene in the regulation of apoptosis (Fitzwalter and Thorburn, 2018), and the activity of *FOXO3* is regulated by the phosphorylation of *AMPK* (Greer, et al., 2007). Interestingly, in the sequencing results, both of them were significantly down-regulated in hTCs and enriched in AMPK signaling pathway. These results hinted that *miR-202-5p* may through targeting *CHPT1* to affect the expression levels of *CCN1* and *FOXO3* under the AMPK pathway, thereby affecting the proliferation of TCs. Previous studies have also shown that *CHPT1* could inhibit the expression of *p53* (Zhuang, et al., 2019), and *p53* can block the activation of *SREBP-2* by *ABCA1*, thereby affecting CH synthesis (Moon, et al., 2019). CH is the precursor of steroid hormones, and its level is closely related to steroid metabolism (Goicoechea, et al., 2023). These results suggested that *miR-202-5p* might a key to regulate the proliferation/apoptosis and lipid/steroid metabolism of TCs during the goose follicle selection, and may affect proliferation and apoptosis by targeting *CHPT1* to regulate *CCN1*/*FOXO3* expression levels; as well as regulate lipid/steroid metabolism and proliferation/apoptosis by targeting *CHPT1* to regulate the *p53*/*ABCA1*/*SREBP-2* expression levels.

## CONCLUSIONS

**Figure 7:**
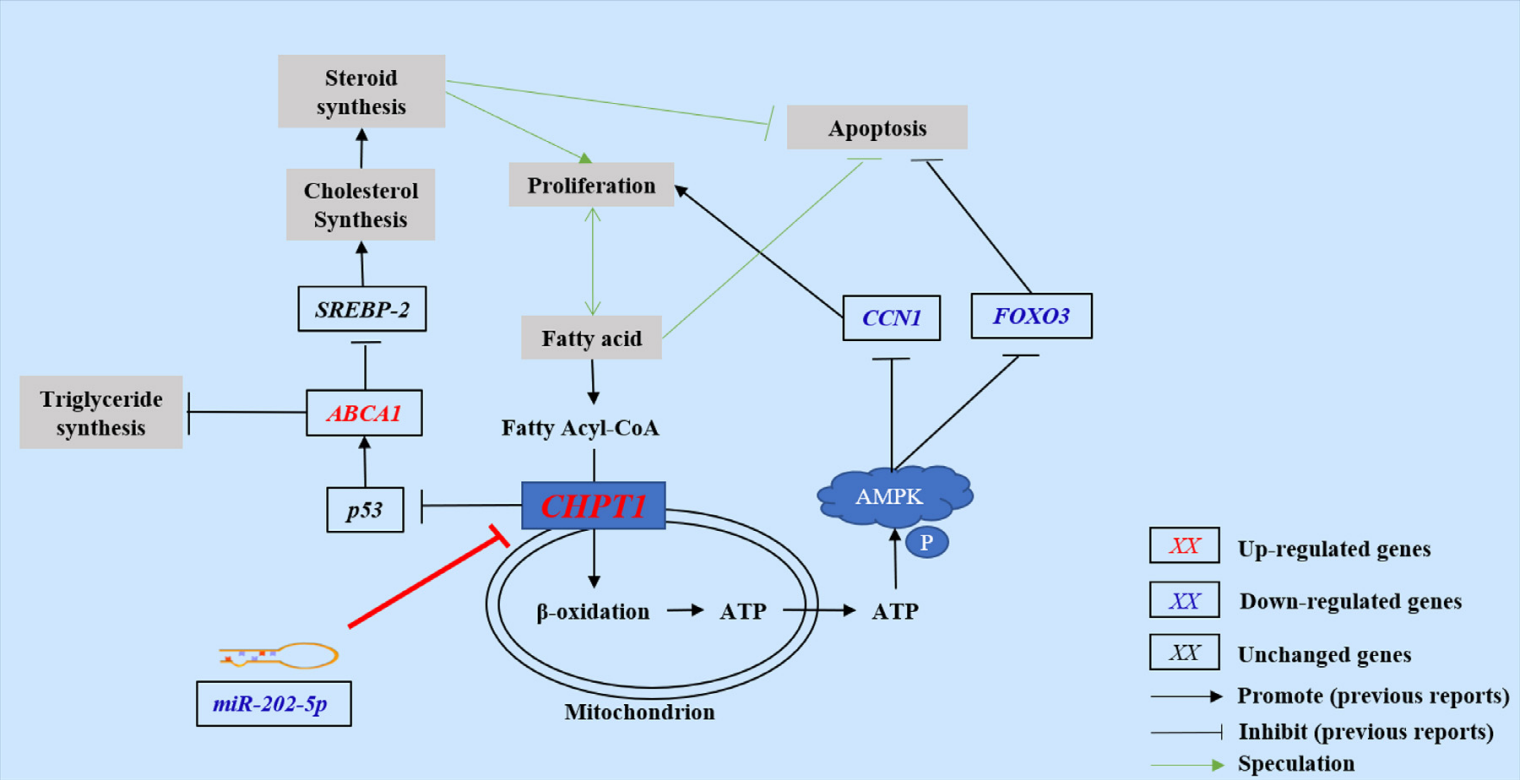
Molecular mechanism prediction of miR-202-5p regulating TCs physiological differences in goose follicle selection stage. Abbreviations: TCs: theca cells; *ABCA1*: ATP binding cassette subfamily A member 1, *CHPT1*: Choline phosphotransferase 1; *CCN1*: Cellular communication network factor 1; *FOXO3*: Forkhead box O3; *SREBP-2*: Sterol regulatory element-binding protein 2.

In this study, significant differences were found in the physiological functions including proliferation/apoptosis and lipid/steroid metabolism between phTCs and hTCs. Then, a total of 1,559 DEGs and 71 DEMs between phTCs and hTCs were identified. Additionally, 15 key DEMs and 17 key DEGs were screened based on functional analysis. Among them, *miR-202-5p* could stably regulate the physiological function of TCs in vivo and vitro. After target gene prediction and functional analysis, it was speculated that *miR-202-5p* may regulate *CCN1/FOXO3* by targeting *CHPT1* to affect TCs proliferation/apoptosis, and may regulate *p53*/*ABCA1*/*SREBP-2* by targeting *CHPT1* to affect TCs lipid/steroid (Figure 7). These results provide new insights into the mechanisms regulating the physiological differences of TCs during goose follicle selection.

## DISCLOSURES

The authors declare no conflicts of interest.

## Appendix Supplementary materials

Image, application 1

Image, application 2

Image, application 3

Image, application 4

Image, application 5

Image, application 6

## Footnotes

Supplementary material associated with this article can be found, in the online version, at doi:10.1016/j.psj.2024.104402.
