# Supplementary material for: Combined analyses of mRNA and miRNA transcriptome reveal the molecular mechanisms of theca cells physiological differences in geese follicular selection stage
Source: Poult Sci. 2024 Oct 10;103(12):104402. doi: 10.1016/j.psj.2024.104402 (PMC11577227; doi:10.1016/j.psj.2024.104402)
Supplement: Supplementary file 1 [file mmc1.docx]

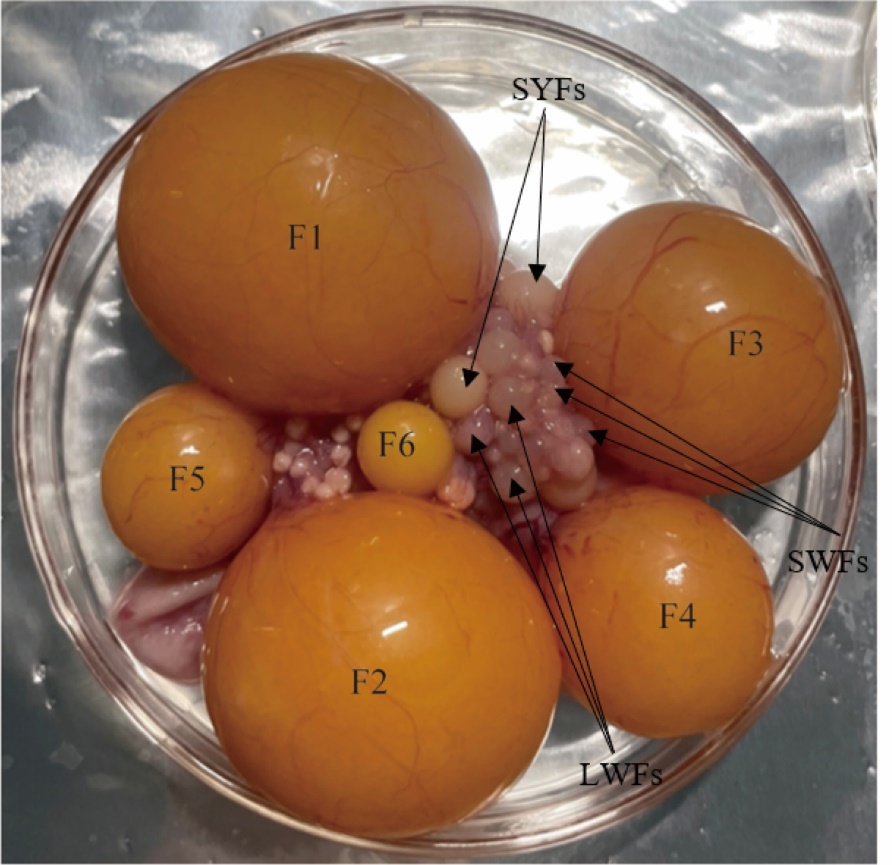


***Supplementary Figure S1. The macroscopic morphology of follicles in Tianfu meat-type geese.*** The follicles include pre-hierarchical (SWFs, LWFs, and SYFs) and hierarchical follicles (F6, F5, F4, F3, F2, and F1). Abbreviations: SWFs: small white follicles, 2 ~ 6 mm in diameter; LWFs: large white follicles, 6 ~ 8 mm in diameter; SYFs: small yellow follicles, 8 ~ 10 mm in diameter.
