# Supplementary material for: Combined analyses of mRNA and miRNA transcriptome reveal the molecular mechanisms of theca cells physiological differences in geese follicular selection stage
Source: Poult Sci. 2024 Oct 10;103(12):104402. doi: 10.1016/j.psj.2024.104402 (PMC11577227; doi:10.1016/j.psj.2024.104402)
Supplement: Supplementary file 2 [file mmc2.xlsx]

***Supplementary Table S1. The information of primers****.*

| **Gene name** | **Sequence ( 5'-3')** | **Length of product (bp)** | **Tm (℃)** |
| --- | --- | --- | --- |
| *GAPDH* | F: TTTCCCCACAGCCTTAGCA | 90 | 60.0 |
|  | R: GCCATCACAGCCACACAGA |  |  |
| *CHPT1* | F: AGTGGTGGCTCACATGACAAA | 192 | 60.0 |
|  | R: TGAGCAGCGATCTGTAGGCA |  |  |
| *FOXO3* | F: CCGCTTGTCACCGATTTTGG | 119 | 60.0 |
|  | R: ACGGTTTGTTTACCGAGGGG |  |  |
| *CCN1* | F: GGCATCTCCACGAGGGTTAC | 200 | 60.0 |
|  | R: TTGGGGCGGTACTTCTTCAC |  |  |
| *ABCA1* | F: GTCAAGCTCCAGCAGGTCAT | 163 | 60.0 |
|  | R: CGGCTGCAAGGAGTTTTTCC |  |  |
| *U6* | F: CAGGTCCAGTTTTTTTTTTTTTT | \ | 55.0 |
|  | R: TACAGAGAAGATTAGCATGG |  |  |
| *miR-202-5p* | F: GCGCTTTCCTATGCATATACT | \ | 55.0 |
|  | R: CAGGTCCAGTTTTTTTTTTTTTT |  |  |
